# Pilot Study: Immune Checkpoints Polymorphisms in Greek Primary Breast Cancer Patients

**DOI:** 10.3390/biomedicines10081827

**Published:** 2022-07-29

**Authors:** Nyanbol Kuol, Xu Yan, Vanessa Barriga, Jimsheena Karakkat, Stamatis Vassilaros, Ioannis Fyssas, Anastasios Tsimpanis, Sarah Fraser, Kulmira Nurgali, Vasso Apostolopoulos

**Affiliations:** 1 Institute for Health and Sport, Victoria University, Melbourne, VIC 3030, Australia; nkuol@med.unr.edu (N.K.); sean.yan@vu.edu.au (X.Y.); vanessa.barriga@live.vu.edu.au (V.B.); jimsheena.valiyakathkarakkat@vu.edu.au (J.K.); sarah.fraser@vu.edu.au (S.F.); kulmira.nurgali@vu.edu.au (K.N.); 2 Department of Physiology and Cell Biology, Reno School of Medicine, University of Nevada, Reno, NV 89557-0352, USA; 3 Prolipsis Medical Diagnostic Centre, 11528 Athens, Greece; stamatisvassilaros@gmail.com (S.V.); jpfyssas@gmail.com (I.F.); helaca@hol.gr (A.T.); 4 Regenerative Medicine and Stem Cells Program, Australian Institute for Musculoskeletal Science (AIMSS), Melbourne, VIC 3021, Australia; 5 Department of Medicine Western Health, Faculty of Medicine, Dentistry and Health Sciences, The University of Melbourne, Melbourne, VIC 3021, Australia; 6 Immunology Program, Australian Institute for Musculoskeletal Science (AIMSS), Melbourne, VIC 3021, Australia

**Keywords:** breast cancer, subtypes, luminal A and B, immune checkpoint inhibitors, single nucleotide polymorphisms

## Abstract

Background: Breast cancer is the most prevalent and second leading cause of cancer-related death in women worldwide. Despite early detection and better treatment therapies, 30% of early-stage breast cancer patients still develop recurrent disease. Breast cancer is a heterogeneous disease comprising several molecular subtypes, commonly classified into clinical subtypes based on the hormone receptor status. These subtypes included luminal A and luminal B, which have different prognoses. Breast cancer development and progression involve many factors. Polymorphisms of PD-1, PD-L1, and PD-L2 genes have been previously associated with high risk and prognosis of cancer. However, no studies have associated PD-1, PD-L1, and PD-L2 polymorphisms with primary breast cancer subtypes. Hence, this study evaluated functional single nucleotide polymorphisms of PD-1, PD-L1, and PD-L2 with primary breast cancer subtypes, luminal A, and luminal B. In addition, we evaluated the PD-L1 protein expression in relation to primary breast cancer subtypes and stages. Results: There were no significant differences in the allele frequencies of PD-1 polymorphisms (rs2227981 G>A, rs7421861 A>G, and rs11568821 C>T) and PD-L1 polymorphisms (rs10815225 C>T and rs2282055 T>G) when compared with the general European population. However, a significant difference was detected in one of the PD-L2 polymorphisms (rs1009759 A>G), with the G allele higher in breast cancer patients than in the general European population. A higher prevalence of the T allele of PD-L1 polymorphism rs2282055 T>G was observed in luminal B breast cancer patients compared with luminal A. No significant difference was detected in other polymorphisms. We also observed that the PD-L1 rs2282055 TT genotype was more prevalent in luminal B breast cancer patients compared with luminal A. Our results found no association of the selected SNPs in the *PDCD1* gene with breast cancer risk. Similarly, the protein expression data showed that PD-L1 and PD-L2 are associated with an aggressive phenotype, Luminal B, and advanced breast cancer stage. Conclusion: These findings suggest that immune checkpoint polymorphisms are associated with the risk and subtypes of breast cancer.

## 1. Introduction

Breast cancer is the most common and second leading cause of cancer-related death in women worldwide [1,2]. Overall, 1 in 8 women worldwide will develop breast cancer, and 1 in 37 women will die before age 85. Although early detection and better treatment regimens for breast cancer have enormously improved patients’ survival outcomes, 30% of early-stage breast cancer patients still develop recurrent disease [3]. Breast cancer is a heterogeneous disease comprising several molecular subtypes, commonly classified into clinical subtypes based on the hormone receptor status [4]. These subtypes include but are not limited to luminal A and luminal B, which have different prognoses [5,6]. Luminal A breast cancer is categorized by a lower expression of estrogen receptor (ER) and progesterone receptor (PR) and a high histologic grade with better overall survival and disease-free survival [7,8]. On the other hand, Luminal B accounted for nearly 40% of all breast cancers classified by aggressive clinical behavior and a high ER and PR [9,10].

The interaction of programmed death-1 (PD-1) with programmed death-ligand 1 (PD-L1) plays a vital role in tumour cell evasion from host immune surveillance by downregulating T cell immune response [11]. The binding of PD-1 to its ligands PD-L1 and/or PD-L2 is responsible for T cell proliferation, activation, and cytotoxic secretion in cancer to disintegrate anti-tumour immune responses [11]. PD-1, also known as CD279, encoded by the *PDCD1* gene, is a cell surface immunosuppressive receptor expressed on immune cells [12,13]. Studies have reported that single nucleotide polymorphisms (SNPs) in the *PDCD-1* gene can influence cancer risk [14,15]. Meta-analysis studies have shown that PD-1 *rs2227981*, *rs36084323* and *rs11568821* polymorphisms are associated with decreased overall cancer risk [16,17,18,19], while *rs7421861* significantly augmented the risk of cancer [16]. However, another meta-analysis found that *rs11568821* is associated with increased risk [14] and no association of *rs2227981* with the risk of cancer [20]. PD-1 *rs2227982* was significantly correlated with age and tumour size, while PD-1 *rs11568821* and *rs2227981* were associated with tumour stage and tumour grade, respectively [19].

PD-L1, also identified as B7-H1 encoded by the *CD274* gene, is a co-inhibitory molecule expressed on activated immune cells, by which cancer cells utilised to evade the host’s immune response [12,21]. Accumulated evidence has shown that PD-L1 expression is induced by IFN-γ; however, microRNAs that control the *PD-L1* gene by binding to its 3 prime untranslated region (3′UTR) have been reported to play a role in controlling PD-L1 expression [22,23,24]. For example, loss of the 3′UTR in the *PD-L1* gene enhances PD-L1 mRNA stability in murine and human murine cells [25]. Furthermore, cells expressing high levels of PD-L1 were noted to have deleted 3′UTR in the *PD-L1* gene [26].

The G allele of PD-L1 *rs7866740* was associated with the risk of non-small lung cell carcinoma (NSCLC) compared to the C allele [27]. Moreover, PD-L1 *rs2890658* C>A and *rs822336* G>C polymorphisms were associated with worse overall survival and progression-free survival of NSCLC patients [27]. Similarly, PD-L1 *rs2890658* A>C genotype and A allele increased the risk of breast cancer [19]. PD-L1 *rs2890658* significantly reduced the risk of lung and liver cancer in the allele model A>C but increased the risk of hepatocellular carcinoma in the allele model A>C and recessive genetic model AA> AC+CC [14]. *PD-L1 s4143815* is associated with a reduced risk of breast cancer (G>C) and hepatocellular carcinoma in homozygote (CC>GG), heterozygote (GC> GG), and dominant (CC+CG >GG) genetic patterns [14]. *PD-L1* rs2890658 and rs4143815 were correlated with age [19]. These findings suggest that PD-L1 polymorphisms could affect the risk and prognosis of cancer.

On the other hand, the role of protein expression of PD-L2 and polymorphism in cancer is not well studied. High expression of PD-L2 was associated with a poor prognostic in patients diagnosed with hepatocellular carcinoma, while no association was observed in patients diagnosed with gastric, renal cell carcinoma, and esophageal cancer [28]. Similarly, PD-L2 protein expression and polymorphism were found to have no significant effect on gastric cancer [29].

Although polymorphisms of PD-1, PD-L1, and PD-L2 genes have been reported to associate with a high risk of cancer, to the best of our knowledge, no studies have reported PD-1, PD-L1, and PD-L2 polymorphisms in relation to primary breast cancer subtypes. Hence, this study evaluated functional single nucleotide polymorphisms of PD-1, PD-L1, and PD-L2 in relation to primary breast cancer subtypes, luminal A, and luminal B. In addition, we evaluated the PD-L1 protein expression in relation to primary breast cancer subtypes and stages.

## 2. Materials and Methods

### 2.1. Human Breast Cancer Samples

Human breast cancer samples were collected at the Prolipsis Medical Centre, Athens, Greece between 2007–2012 (ethics approved 12 June 2006). The human samples collection used in this study complied with the guidelines of the National Health and Medical Research Council (NHMRC) Australian Code of Practice for the Care under the approval of the Victoria University Human Ethics Committee (ethics number HREC15-299). All patients signed written informed consent to use their tissues for research purposes. None of the breast cancer patients had a second neoplastic disease or had previously undergone chemo- or radiotherapy. All samples were coded. To achieve a significantly different, a power of 0.8, two-tails, and alpha = 0.05 with two subtypes, medium effect size, F = 0.30 was used to calculate sample size using GPOWER. A minimum of 82 samples (minimum of *n* = 41/subtypes) were required to achieve a significantly different. In this study, 123 patients with breast cancer (81 patients had luminal A, and 42 had luminal B) were analysed for SNP, and 74 (subtypes, luminal A *n* = 43, luminal B *n* = 16; stage I *n* = 26, stage II *n* = 19, and stage III *n* = 14) for immunofluorescences. Immunofluorescence images were quantified blindly. Samples were fresh-frozen and stored at −80 °C.

### 2.2. DNA Extraction from Fresh-Frozen Samples

DNA extraction from fresh-frozen samples of patients diagnosed with primary breast cancer was performed according to Kurabo DNA extraction kits (Kurabo, Japan). Briefly, samples were incubated overnight with tissue lysis buffer and proteinase K at 55 °C with a rotary shaker. Samples were centrifuged at 10,000 revolutions per minute for 3 min (mins), and the supernatant was transferred to a new microtube. Samples were treated with RNase A, vortexed for 5 s (sec), and flash spun before incubation for 2 min at room temperature. Then, samples were incubated with lysis buffer at 70 °C for 10 min with a rotary shaker. Samples were flash spun, 99% ethanol was added, they were vortexed for 15 sec and flash spun. Lysates were transferred into the cartridge of QuickGene, and washes were performed using wash buffer. Cartridges were incubated with elution buffer for 90 sec at room temperature, and genomic DNA was collected into a new tube. Genomic DNA was run through electrophoresis gel to check purity and qubit to quantify genomic DNA concentration. Genomic DNA was then sent off to the Australian Genome Research Facility (AGRF, Brisbane, QLD, Australia) for SNPs analysis using MassARRAY^®^ on a Compact Spectrometer.

### 2.3. SNP Selection

Three SNPs (rs2227981, rs7421861, and rs11568821) in PD-1, two (rs10815225 and rs2282055) in PD-L1, and two (rs1009759 and rs6476985) in PD-L2 genes were selected based on previously published literature associated with cancer or other diseases. The control data were obtained from the European subgroup of ALFA: Allele Frequency Aggregator (ALFA: Allele Frequency Aggregator National Center for Biotechnology Information, U.S. National Library of Medicine, www.ncbi.nlm.nih.gov/snp/docs/gsr/alfa/ (accessed on 10 March 2020)).

### 2.4. Immunofluorescence

Tumour tissues were fixed in 10% formalin for 24 h, rinsed with 70% ethanol, and paraffin-embedded. Serial sections of 10 µm were cut from each sample and were mounted on immunohistochemistry microscope glass slides. All slides were deparaffinized with xylene and rehydrated with graded ethanol series before being submerged in citrate buffer (pH 6) (Sigma-Aldrich, Melbourne, VIC, Australia), then placed on a pre-heated hot plate set at 100 °C for 20 min and left to cool at room temperature for another 20 min. Slides were washed with phosphate-buffered saline 3 × 10 min. To reduce the volume, samples were outlined using a liquid blocker super pap pen (ProSciTech). The endogenous activity was blocked by incubating slides with 10% donkey serum for 1 h at room temperature. Slides were then incubated overnight with target primary antibodies against PD-L1 (1:500, Abcam, ab210931) [30,31] and PD-L2 (1:500, Abcam, ab200377) [32,33], followed by Alexa Fluor- 594 conjugated secondary antibodies (Abacus, JI711585152) diluted 1:250 in phosphate-buffered saline containing 2% donkey serum and 0.01% Triton X-100. Samples were incubated for 1 min with 4,6-Diamidine-2-phenylindole dihydrochloride (DAPI) (D1306, Life Technologies Australia Pty Ltd.) and mounted using DAKO mounting media and coverslips applied. Anti-PD-L1 and anti-PD-L2 antibodies were validated using colon cancer tissues which expressed high levels of PD-L1 and PD-L2. PD-L1 was noted to be expressed in mucosa and muscularis mucosal layers, whereas PD-L2 was predominantly expressed in mucosa with the exception to stage IV, where it was expressed in both layers (Kuol et al., submitted for publication).

### 2.5. Data Analysis

SNP analysis: Hardy–Weinberg equilibrium (HWE) was calculated by AGRF. Fisher’s exact test was conducted by GraphPad Prism for the relationship of PD-1, PD-L1, and PD-L2 morphisms allele frequencies with luminal A and B. Chi-square test (χ^2^) was conducted by GraphPad Prism for the relationship of PD-1, PD-L1, and PD-L2 morphisms genotypes with luminal A and B. Statistical analysis of immunohistochemical data was performed by Student’s *t*-test and one-way ANOVA. Statistical significance was defined as *p* < 0.05 (two-tailed).

Immunofluorescences analysis: Images were captured on a Nikon Eclipse Ti multichannel confocal laser scanning system. Z-series images were acquired at a nominal thickness of 1 μm (1024 × 1024 pixels). Image J software (NIH, Bethesda, MD, USA) was employed to convert RGB images to greyscale 8-bit binary; particles were then analysed to obtain the percentage area of immunoreactivity [34]. All slides were coded, and immunohistochemistry images were quantified blindly.

## 3. Results

### 3.1. Clinicopathological and Demographic Parameters of Breast Cancer Patients

The clinicopathological and demographic parameters of breast cancer patients are presented in Table 1. The average patient’s age was 65 years ranging from 30 to 86, 75 (61%) patients were below the age of 65, and 48 (39%) were above 65 years of age in the cohort. Among these patients, 74 (60.2%) patients had a tumour size less than 2 cm, and 49 (39.8%) had a tumour size greater than 2 cm. In this cohort, 3 (2.4%) patients were diagnosed with clinical stage 0, 59 (48%) with stage I, 33 (26.8%) with stage II, and 19 (15.4%) with stage III breast cancer; 9 (7.3%) has missing stage data. This study comprised 123 patients with breast cancer; 81 (65.9%) patients had luminal A, and 42 (34.1%) had luminal B.

The distribution of genotype frequencies in controls accorded with Hardy-Weinberg equilibrium (HWE) (Table 2 and Table 3). For PD-1 polymorphisms (rs2227981 G>A, rs7421861 A>G and rs11568821 C>T), the genotyping success rate was more than 92% (Table 2). For PD-L1polymorphisms (rs10815225 C>T and rs2282055 T>G) and PD-L2 polymorphisms (rs1009759 A>G and rs6476985 C>T), the genotyping success rate was more than 94% (Table 3).

### 3.2. Association of PD-1, PD-L1, and PD-L2 Polymorphisms with Breast Cancer

The allele frequencies of PD-1, PD-L1, and PD-L2 polymorphisms for breast cancer patients are summarized in Table 4, together with the allele frequencies in the European population. There were no significant differences in the allele frequencies of PD-1 polymorphisms (rs2227981 G>A, rs7421861 A>G, and rs11568821 C>T) between the breast cancer and the general European population (Table 4, *p* > 0.05). Similarly, no significant differences were detected for the PD-L1 polymorphisms (rs10815225 C>T and rs2282055 T>G) (Table 4, *p* > 0.05). A significant difference was detected in one of the PD-L2 polymorphisms (rs1009759 A>G), with the G allele higher in the breast cancer patients when compared with the general European population (Table 4, *p* = 0.038).

### 3.3. Association of PD-1 PD-L1 and PD-L2 Polymorphisms with Cancer Subtypes

We then compared the allele frequencies of those polymorphisms in two common breast cancer subtypes, luminal A and luminal B. We observed a higher prevalence of the T allele of PD-L1 polymorphism rs2282055 T>G in luminal B breast cancer patients (Table 5, *p* = 0.022). Since luminal B is more aggressive, our results demonstrated that rs2282055 T>G is associated with breast cancer severity. However, no significant differences were detected in other polymorphisms.

We then compared the genotype frequencies of those polymorphisms in the two common breast cancer subtypes. Due to the relatively small sample size, our statistical analyses were not valid for PD-1 polymorphism rs11568821 C>T or PD-L1 polymorphisms (rs10815225 C>T (Table 6). However, we did find a significant difference in the genotype distribution of PD-L1 polymorphism rs2282055 T>G) between luminal B and luminal A breast cancer patients (Table 6, *p* = 0.049).

We then applied different genetic models to our analyses. We observed a significant difference in PD-L1 rs2282055 TT vs. GG genotype frequencies between luminal B and luminal A breast cancer patients, with the TT genotype more likely to develop luminal B breast cancer (Table 7, *p* = 0.044). When we combined the TT genotype with the TG genotype, we observed a higher presence in luminal B breast cancer patients when compared with the GG genotype (Table 7, *p* = 0.051). We also found a significant difference when PD-1 polymorphism rs7421861 A>G AA+AG genotype frequencies were compared with GG between luminal B and luminal A breast cancer patients (Table 7, *p* = 0.042). The GG genotype appears more prevalent in luminal B breast cancer patients.

### 3.4. Enhanced Expression of PD-L1 and PD-L2 Associates with Luminal B and Advanced Stage in Greek Primary Breast Cancer Cohort

The fact that the results of gene expression studies are not always identical and should not be directly translated into protein expression studies is well-known. Post-transcriptional mechanisms can shape protein levels independently of mRNA abundance [35]. Furthermore, the expression of proteins is closer to the phenotypic changes than the gene expression profiles. In addition, it is well established that tumours can form a microenvironment aiding their growth progression. The microenvironment constituents can influence one another, leading to detrimental effects [36,37]. However, the differences should not underestimate the value of any expression. We speculate that the gene and protein expression could hold a prognostic valuable in breast cancer.

**Figure 1:**
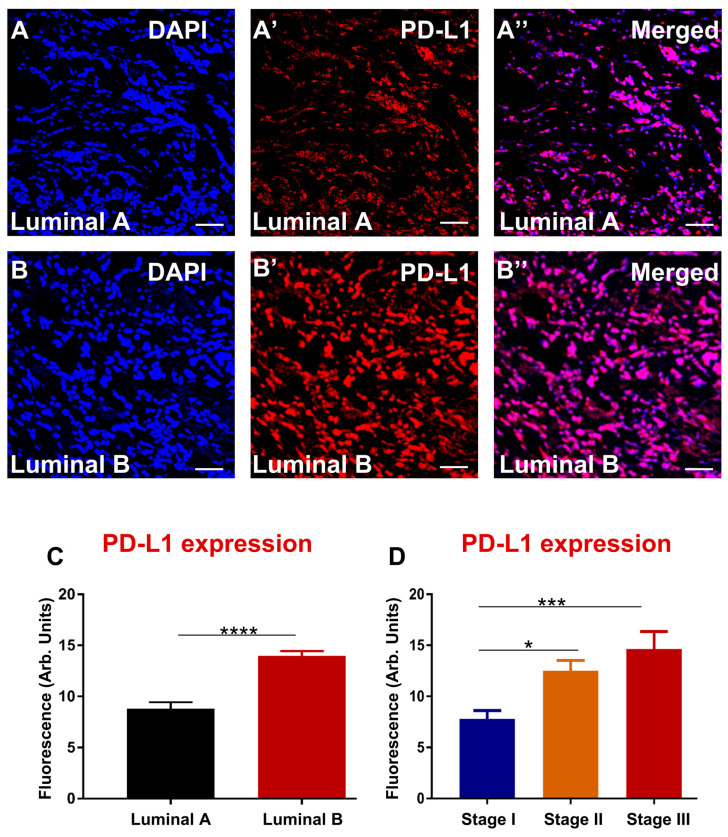
PD-L1 in human specimens from breast cancer patients diagnosed with luminal A (**A**–**A″**) and luminal B (**B**–**B″**). Tissues were labelled with the nuclei marker DAPI (blue; **A**–**B**), PD-L1 (red; **A′**–**B′**), and all markers merged (**A″**–**B″**). Scale bar represents 50µm. Bar graphs displaying the mean fluorescence of PD-L1 (**C**) and PD-L1 correlation with stages of breast cancer (**D**). Data presented as mean ± standard error of the mean (SEM), subtypes, luminal A *n* = 43, Luminal B *n* = 16; stage I *n* = 26, stage II *n* = 19, and stage III *n* = 14. Student’s *t*-test and One-way ANOVA, * *p* < 0.05, *** *p* < 0.001, **** *p* < 0.0001.

**Figure 2:**
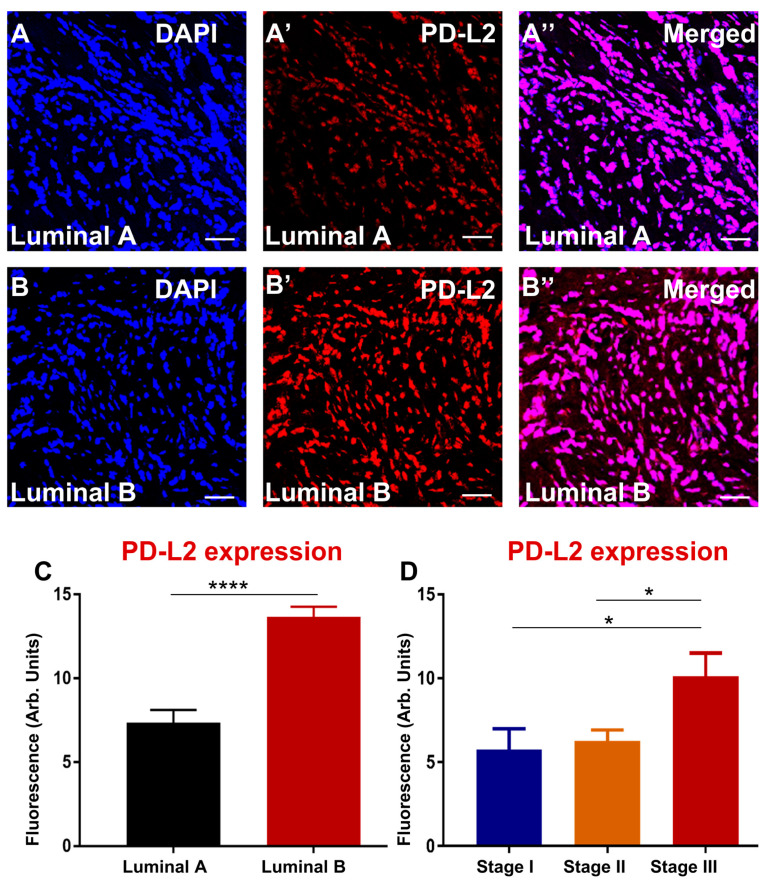
PD-L2 in human specimens from breast cancer patients diagnosed with luminal A (**A**–**A″**) and luminal B (**B**–**B″**). Tumors were labelled with the nuclei marker DAPI (blue; **A**–**B**), PD-L2 (red; **A′**–**B′**), and all markers merged (**A″**–**B″**). Scale bar represents 50µm. Bar graphs displaying the mean fluorescence of PD-L2 (**C**) and PD-L2 correlation with stages of breast cancer (**D**). Data presented as mean ± standard error of the mean (SEM), subtypes, luminal A *n* = 25, Luminal B *n* = 16; stage I *n* = 17, stage II *n* = 17, and stage III *n* = 7. Student’s *t*-test and One-way ANOVA, * *p* < 0.05, **** *p* < 0.0001.

Our SNPs data showed that PD-L1 polymorphisms are associated with an aggressive subtype, luminal B. To determine whether, at the protein level in the tumour samples, PD-L1 expression is more abundant in luminal B, immunofluorescence was used. Due to the nature of this study being a blinded pilot study, tumour samples analysed for the subtype and stage were not the same “n” number. All samples were coded, and immunohistochemistry images were quantified blindly. The results showed that overexpression of PD-L1 was associated with luminal B compared with luminal A (Figure 1A–C). In addition, advanced stage III of breast cancer abundantly expressed PD-L1 compared to lower stages I and II (Figure 1D). Moreover, a significant difference was observed between stage II and stage I. There seems to be a linear relationship between stage progression and PD-L1 expression. Although PD-L2 polymorphism was not associated with breast cancer severity, high expression of PD-L2 was associated with luminal B subtype and advanced stage III compared to early stages I and II (Figure 2A–D). Overall, enhanced PD-L1 and PD-L2 levels in breast cancer tissues are associated with clinical parameters that correlate with poor patient prognosis.

Taken together, our protein expression data were consistent with SNP data, suggesting that both PD-L1 SNPs and protein expression hold prognostic and predicted values for the breast cancer subtype.

## 4. Discussion

The ability of cancer cells to evade T cell responses and avoid immune recognition by disabling effector T cells is dependent on the multiple immunosuppressive mechanisms controlled by immune checkpoints of inhibitory pathways, including but not limited to PD-L1 and PD-L2. These immune checkpoints are initiated by ligand-receptor interactions, some of which have been FDA approved to enhance anti-tumour immunity [38,39]. The expression of PD-L1 and PD-L2 has suggested the importance of other interactions between the tumour and host immune system that allow cancer cells to evade the immune responses leading to tumour growth. Currently, breast cancer research has focused on the factors that influence the growth and spread of cancer cells in immune evasion mechanisms [40]. Although these studies have provided evidence of the PD-L1 role in breast cancer, there is limited knowledge associating PD-L1 protein expression and polymorphism with breast cancer patients’ clinical parameters, such as subtype and stage. Thus, in the present study, we performed a correlation analysis of neuro-immune markers in breast cancer tumours categorized by subtype and stage.

To the best of our knowledge, this study is the first to determine immune checkpoint polymorphisms and their correlation to the stage and subtype of breast cancer. To assess the polymorphism of PD-1, PD-L1, and PD-L2 and their correlation to breast cancer subtype, primary tumour samples were obtained from breast cancer patients, and tumours were defined into subtypes based on the expression of clinically used markers ER, PR, and HER2; and stage I, II, and III. For protein expression, tumour tissues were immunolabelled with PD-L1 and PD-L2 antibodies described under materials and methods.

PD-1, PD-L1, and PD-L2 polymorphisms are selected based on previous publications, indicating their association with cancer risk. Out of the seven polymorphisms, only the PD-L2→rs1009759 A>G polymorphism showed a significant difference between the breast cancer patients and the European population. PD-L2 rs1009759 A>G polymorphism has not been studied extensively in the literature. Only one study has checked its association with ankylosing spondylitis and reported no association with the disease [41]. When checking the PD-L2 rs1009759, A>G polymorphism with breast cancer subtypes, neither allele frequency nor genotype frequency showed any correlation, likely due to the small sample size.

On the other hand, several polymorphisms showing no difference between breast cancer patients and the general European population, including PD-1 rs7421861 A>G and PD-L1 rs2282055 T>G, have been correlated with breast cancer subtypes. Out of 40 luminal B breast cancer patients, the T allele of rs2282055 is more prevalent with no GG phenotype. A 2017 study reported that the G allele of PD-L1 rs2282055 was associated with better clinical response compared with the T allele in non-small-cell lung cancer patients [42]. Since luminal B breast cancer subtype has a worse clinical outcome when compared with luminal A subtype, we have reason to believe the G allele and GG phenotype are associated with better clinical outcomes in breast cancer patients. The PD-1 rs7421861 is a relatively more studied polymorphism associated with colorectal cancer risk [43]. Similar to our results, rs7421861 polymorphism frequency was not associated with breast cancer risk in the Chinese population [44,45]. A meta-analysis also showed no correlation between rs7421861 polymorphism frequency and cancer risk [18]. Unfortunately, no study has the clinical response among cancer patients with different rs7421861 alleles or genotypes. Our results show that the rs7421861 GG genotype is more prevalent in the luminal B subtype breast cancer patients, suggesting that the GG genotype is associated with worse clinical outcomes.

Cancer cells use multiple mechanisms to avoid being recognized by the immune system and downregulate the expression of PD-L1 on their surface, interacting with PD-1 on tumour-infiltrating lymphocytes [46]. PD-L1 on the surface of cancer cells functions as an immune resistance mechanism, allowing cancer cells to go undetected and leading to proliferation and rapid advancement [21]. In contrast, several studies have demonstrated that the expression of PD-L1 on immune cells has a favorable prognostic factor in some cancers [47]. Increased expression of PD-L1 on cancer cells has been used in clinical trials to identify patients that will benefit from immunotherapy. Moreover, a decreased expression of PD-L1 in subsets of breast cancer patients has also shown a clinical benefit [38,48,49,50]. This study evaluated the expression of PD-L1 in different subtypes of breast cancer. The results demonstrated upregulation of PD-L1 expression in luminal B subtype compared to luminal A. PD-L1 expression in the epithelium and stroma associated with triple-negative hormone receptor expression reported in triple-negative breast cancer [51]. The expression of PD-L1 in TNBC was associated not only with cancer but identified in TILs [52]. Furthermore, the enhanced expression of PD-L1 was associated with stage III of breast cancer patients compared to stage I and II. These data correlate with studies that show advanced clinical stage and higher tumour grade with the high expression of PD-L1 [53].

The role of PD-L2 and its prognostic significance in cancer remains understudied. Some studies have associated high expression of PD-L2 with poor patient survival [28,54,55,56]. High PD-L2 was associated with worse patient survival outcomes, as noted in hepatocellular carcinoma [28,54], colorectal cancer [55], and renal cell carcinoma [56]. Although we did not correlate the expression of PD-L2 with patient survival outcomes, our findings did associate high levels of PD-L2 with severe breast cancer subtype and advanced stage.

There are a couple of limitations to be acknowledged. First, the control group’s data were obtained from the European population of the ALFA study, which might not represent the exact genetic makeup of the PD-1, PD-L1, and PD-L2 polymorphisms in the Greek population. Secondly, the control population includes males and females; while the present study patients are exclusively females, there might be different allele frequencies between males and females. Lastly, our sample size is relatively small among cancer risk studies. Nevertheless, the fact that the PD-1 rs7421861 A>G and PD-L1 rs2282055 T>G polymorphisms correlate with the severity of cancer probably suggests that these polymorphisms are associated with clinical outcomes among breast cancer patients.

## 5. Conclusions

The presence of PD-L1 and PD-L2 on the surface of cancer cells functions as an immune resistance mechanism, allowing them to go undetected and leading to cancer cell proliferation and progression of cancer growth. Studies have reported the expression of these markers at protein levels and their relevance to prognostic outcomes. However, there are limited studies on these markers’ polymorphisms. Therefore, revealing the significance of these markers’ polymorphisms in cancer is imperative for understanding breast cancer development and progression.

## Figures and Tables

**Table 1:** Clinicopathological and demographic parameters of breast cancer patients.

Parameters	No. of Cases	Percentage (%)
Total	123	100
Tumour size		
<2	74	60.2
>2	49	39.8
Age		
<65	75	61
>65	48	39
Stage		
0	3	2.4
I	59	48
II	33	26.8
III	19	15.4
Missing	9	7.3
Subtype		
Luminal A	81	65.9
Luminal B	42	34.1

**Table 2:** Primary information for PD-1 polymorphisms (rs2227981 G>A, rs7421861 A>G and rs11568821 C>T).

	rs2227981 G>A	rs7421861 A>G	rs11568821 C>T
Chromosome	2	2	2
Position	241851121	241853198	241851760
Region	2KB Upstream	Intron	Intron
MAF in Europeans	0.4013	0.3517	0.1086
*p*-Value for HWE	0.20	0.88	0.52
APR	92.75%	96.38%	98.70%

**Table 3:** Primary information for PD-L1 polymorphisms (rs10815225 C>T and rs2282055 T>G) and PD-L2 polymorphisms (rs1009759 A>G and rs6476985 C>T).

	rs10815225 G>C	rs2282055 T>G	rs1009759 A>G	rs6476985 C>T
Chromosome	9	9	9	9
Position	5450497	5455732	5556786	5517559
Region	2KB Upstream	Intron	Intron	Intron
MAF in Europeans	0.1347	0.2605	0.3101	0.1126
*p*-Value for HWE	0.07	0.82	0.99	0.86
APR	98.55%	98.55%	94.38%	98.70%

**Table 4:** The allele frequencies of PD-1 polymorphisms (rs2227981 G>A, rs7421861 A>G, and rs11568821 C>T), PD-L1 polymorphisms (rs10815225 C>T and rs2282055 T>G), and PD-L2 polymorphisms (rs1009759 A>G and rs6476985 C>T) in breast cancer patients and the European population.

Genes	Alleles	Breast Cancer		European Population		OR Caner to Population (95% CI)	*p*
		*n*	%	*n*	%		
PD-1	rs2227981 G>A						
	G	142	55.47%	5600	59.87%	1.20 (0.93–1.54)	0.175
	A	114	44.53%	3754	40.13%		
	rs7421861 A>G						
	A	184	69.17%	61,022	64.83%	1.22 (0.94–1.58)	0.157
	G	82	30.83%	33,102	35.17		
	rs11568821 C>T						
	C	247	90.15%	10,322	89.14%	1.12 (0.75–1.66)	0.694
	T	27	9.85%	1258	10.86%		
PD-L1	rs10815225 G>C						
	G	230	84.56%	1793	86.53%	1.17 (0.82–1.67)	0.398
	C	42	15.44%	279	13.47%		
	rs2282055 T>G						
	T	204	75.00%	28,314	73.95%	0.95 (0.72–1.25)	0.729
	G	68	25.00%	9972	26.05%		
PD-L2	rs1009759 A>G						
	A	165	62.98%	50,016	68.99%	0.77 (0.60–0.98)	0.038 *
	G	97	37.02%	22,478	31.01%		
	rs6476985 C>T						
	C	239	87.23%	34,559	88.74%	0.87 (0.61–1.25)	0.442
	T	35	12.77%	4383	11.26%		

*n*, number of samples; PD-1, programmed cell death protein 1; PD-L1, programmed cell death ligand 1; PD-L2, programmed cell death ligand 2, * *p* < 0.05

**Table 5:** The allele frequencies of PD-1 polymorphisms (rs2227981 G>A, rs7421861 A>G, and rs11568821 C>T), PD-L1 polymorphisms (rs10815225 C>T and rs2282055 T>G), and PD-L2 polymorphisms (rs1009759 A>G and rs6476985 C>T) in luminal B and luminal A breast cancer patients.

Genes	Alleles	Luminal B		Luminal A		OR Luminal B to A (95% CI)	*p*
		*n*	%	*n*	%		
PD-1	rs2227981 G>A						
	G	48	63.16%	76	50.67%	1.67 (0.95–2.93)	0.090
	A	28	36.84%	74	49.33%		
	rs7421861 A>G						
	A	51	63.75%	114	72.15%	0.68 (0. 9–1.20)	0.234
	G	29	36.25%	44	27.85%		
	rs11568821 C>T						
	C	71	86.59%	149	91.98%	0.56 (0.24–1.26)	0.254
	T	11	13.41%	13	8.02%		
PD-L1	rs10815225 G>C						
	G	70	87.50%	135	83.33%	1.40 (0.64–2.94)	0.452
	C	10	12.50%	25	16.67%		
	rs2282055 T>G						
	T	66	82.50%	111	68.52%	2.17 (1.10–4.19)	0.022 *
	G	14	17.50%	51	31.48%		
PD-L2	rs1009759 A>G						
	A	54	67.50%	92	59.74%	1.40 (0.79–2.49)	0.259
	G	26	32.50%	62	40.26%		
	rs6476985 C>T						
	C	74	90.24%	140	86.42%	1.45 (0.62–3.24)	0.536
	T	8	9.76%	22	13.58%		

*n*, number of samples; PD-1, programmed cell death protein 1; PD-L1, programmed cell death ligand 1; PD-L2, programmed cell death ligand 2, * *p* < 0.05

**Table 6:** Overall analyses of PD-1 polymorphisms (rs2227981 G>A, rs7421861 A>G, and rs11568821 C>T), PD-L1 polymorphisms (rs10815225 C>T and rs2282055 T>G), and PD-L2 polymorphisms (rs1009759 A>G and rs6476985 C>T) genotypes in luminal B and luminal A breast cancer patients. NV, not valid.

Genes	Genotype	Luminal B		Luminal A		*p*
		*n*	%	*n*	%	
PD-1	rs2227981 G>A					
	GG	17	44.74%	21	28.00%	0.197
	GA	14	36.84%	34	45.33%	
	AA	7	18.42%	20	26.67%	
	rs7421861 A>G					
	AA	18	45.00%	39	49.37%	0.084
	AG	15	37.50%	36	45.57%	
	GG	7	17.50%	4	5.06%	
	rs11568821 C>T					
	CC	32	78.05%	68	83.95%	NV
	CT	7	17.07%	13	16.05%	
	TT	2	4.88%	0	0	
PD-L1	rs10815225 G>C					
	GG	31	77.50%	58	71.60%	NV
	GC	8	20.00%	19	23.46%	
	CC	1	2.50%	4	4.94%	
	rs2282055 T>G					
	TT	26	65.00%	38	46.91%	0.049 *
	TG	14	35.00%	35	43.21%	
	GG	0	0	8	9.88%	
PD-L2	rs1009759 A>G					
	AA	19	47.50%	28	36.37%	0.494
	AG	16	40.00%	36	46.75%	
	GG	5	12.50%	13	16.88%	
	rs6476985 C>T					
	CC	33	80.49%	61	75.31%	0.549
	CT	8	19.51%	18	22.22%	
	TT	0	0	2	2.47%	

*n*, number of samples; PD-1, programmed cell death protein 1; PD-L1, programmed cell death ligand 1; PD-L2, programmed cell death ligand 2, * *p* < 0.05.

**Table 7:** Stratified analyses of PD-1 polymorphisms (rs2227981 G>A, rs7421861 A>G, and rs11568821 C>T), PD-L1 polymorphisms (rs10815225 C>T and rs2282055 T>G), and PD-L2 polymorphisms (rs1009759 A>G and rs6476985 C>T) genotypes in luminal B and luminal A breast cancer patients.

Genes	Genotype	Luminal B		Luminal A		OR Luminal B to A (95% CI)	*p*
		*n*		*n*			
PD-1	**rs2227981 G>A**						
	GG vs. AA	17	7	21	20	2.31 (0.76–6.28)	0.192
	GG+GA vs. AA	31	7	55	20	1.61 (0.60–4.07)	0.362
	GG vs. GA+AA	17	21	21	54	2.08 (0.95–4.56)	0.093
	**rs7421861 A>G**						
	AA vs. GG	18	7	39	4	0.26 (0.08–1.04)	0.084
	AA+AG vs. GG	33	7	75	4	0.25 (0.08–0.89)	0.042 *
	AA vs. AG +GG	18	22	39	40	0.84 (0.38–1.79)	0.700
	**rs11568821 C>T**						
	CC vs. TT	32	2	68	0	0	0.109
	CC+CT vs. TT	39	2	81	0	0	0.111
	CC vs. CT+TT	32	9	68	13	0.68 (0.27–1.66)	0.460
PD-L1	**rs10815225 G>C**						
	GG vs. CC	31	1	58	4	2.14 (0.33–26.91)	0.658
	GG+GC vs. CC	39	1	77	4	2.03 (0.32–25.36)	>0.999
	GG vs. GC+CC	31	9	58	23	1.37 (0.58–3.20)	0.521
	**rs2282055 T>G**						
	TT vs. GG	26	0	38	8	Infinity	0.044 *
	TT+TG vs. GG	40	0	73	8	Infinity	0.051
	TT vs. TG+GG	26	14	38	43	2.10 (0.98–4.53)	0.081
PD-L2	**rs1009759 A>G**						
	AA vs. GG	19	5	28	13	1.76 (0.55–5.17)	0.401
	AA+AG vs. GG	35	5	64	13	1.42 (0.46–3.84)	0.600
	AA vs. AG+GG	19	21	28	49	1.58 (0.72–3.50)	0.320
	**rs6476985 C>T**						
	CC vs. TT	33	0	61	2	Infinity	0.544
	CC+CT vs. TT	41	0	79	2	Infinity	0.550
	CC vs. CT+TT	33	8	61	20	1.35 (0.54–3.35)	0.650

*n*, number of samples; PD-1, programmed cell death protein 1; PD-L1, programmed cell death ligand 1; PD-L2, programmed cell death ligand 2, * *p* < 0.05.
